# Maintaining RNA integrity in a homogeneous population of mammary epithelial cells isolated by Laser Capture Microdissection

**DOI:** 10.1186/1471-2121-11-95

**Published:** 2010-12-06

**Authors:** Claudia Bevilacqua, Samira Makhzami, Jean-Christophe Helbling, Pierre Defrenaix, Patrice Martin

**Affiliations:** 1 INRA, UMR1313 Unité Génétique Animale et Biologie Intégrative, équipe « Lait, Génome & Santé » F-78350 Jouy-en-Josas, France; 2 INRA-Plateforme ICE (Iso Cell Express), F-78350 Jouy-en-Josas, France; 3 Excilone SARL, Microgenomic services, F-78490 Vicq, France; 4 UMR1286 PsyNuGen Unité de Psychoneuroimmunologie, Nutrition et GénétiqueInstitut François Magendie 146 rue Léo Saignat 33077 BORDEAUX CEDEX

## Abstract

**Background:**

Laser-capture microdissection (LCM) that enables the isolation of specific cell populations from complex tissues under morphological control is increasingly used for subsequent gene expression studies in cell biology by methods such as real-time quantitative PCR (qPCR), microarrays and most recently by RNA-sequencing. Challenges are i) to select precisely and efficiently cells of interest and ii) to maintain RNA integrity. The mammary gland which is a complex and heterogeneous tissue, consists of multiple cell types, changing in relative proportion during its development and thus hampering gene expression profiling comparison on whole tissue between physiological stages. During lactation, mammary epithelial cells (MEC) are predominant. However several other cell types, including myoepithelial (MMC) and immune cells are present, making it difficult to precisely determine the specificity of gene expression to the cell type of origin. In this work, an optimized reliable procedure for producing RNA from alveolar epithelial cells isolated from frozen histological sections of lactating goat, sheep and cow mammary glands using an infrared-laser based Arcturus Veritas LCM (Applied Biosystems^®^) system has been developed. The following steps of the microdissection workflow: cryosectioning, staining, dehydration and harvesting of microdissected cells have been carefully considered and designed to ensure cell capture efficiency without compromising RNA integrity.

**Results:**

The best results were obtained when staining 8 μm-thick sections with Cresyl violet^® ^(Ambion, Applied Biosystems^®^) and capturing microdissected cells during less than 2 hours before RNA extraction. In addition, particular attention was paid to animal preparation before biopsies or slaughtering (milking) and freezing of tissue blocks which were embedded in a cryoprotective compound before being immersed in isopentane. The amount of RNA thus obtained from *ca*.150 to 250 acini (300,000 to 600,000 μm^2^) ranges between 5 to 10 ng. RNA integrity number (RIN) was *ca*. 8.0 and selectivity of this LCM protocol was demonstrated through qPCR analyses for several alveolar cell specific genes, including *LALBA *(α-lactalbumin) and *CSN1S2 *(α_s2_-casein), as well as *Krt14 *(cytokeratin 14), *CD3e *and *CD68 *which are specific markers of MMC, lymphocytes and macrophages, respectively.

**Conclusions:**

RNAs isolated from MEC in this manner were of very good quality for subsequent linear amplification, thus making it possible to establish a referential gene expression profile of the healthy MEC, a useful platform for tumor biomarker discovery.

## Background

One of the main challenges biologists currently face is overcoming the problem of tissue heterogeneity to further understand organ function. It is crucial to distinguish which cell populations produce specific molecules or to get relevant expression profiles reflecting in vivo status.

Milk is synthesized in mammary gland during lactation and though this process has been thoroughly studied, we still do not know precisely what mechanisms are involved in the intracellular transport and secretion of milk components, including supra-molecular structures, such as casein micelles [1,2] which are assembled during their transit within the mammary epithelial cell (MEC).

Mammary parenchyma consists of secretory alveoli organized into lobules and interconnected by a system of branching ducts separated from adipocytes by multiple layers of fibroblastic connective tissue. In the duct and alveoli, the mammary epithelium is organized into two layers, a basal layer of myoepithelial cells (MMC) and a luminal layer of MEC that secretes milk [3]. The extra cellular matrix comprises non-epithelial cells: fibroblast, endothelial cells, lymphocytes, adipocytes, neurons, myocytes, etc. Thus, the adult mammary gland during lactation is a complex tissue consisting of several cell types. During lactation, epithelial cells are predominant relative to adipocytes which are conversely more abundant in the nulliparous gland [3]. Since both cell types are involved in lipid metabolism using the same metabolic pathways and enzymes, it becomes difficult to sort out the function of each cell type [4,5].

Advances in molecular biology have provided new tools, including gene expression profiling, to analyze mechanisms controlling mammary gland development and differentiation [6,7] and regulating milk synthesis and secretion. However, most of the studies performed to date on healthy mammary gland have been done without taking into account the complexity of this tissue with the exception of Grigoriadis *et al*. [8]. On the other hand, a number of integrated approaches combining advanced molecular technologies have been applied to analyze human breast cancer [9-11], but few studies were carried out on healthy breast tissue compared to carcinoma [12,13]. Analysis of bulk mammary tissue homogenates leads inevitably to an average measurement of biomolecules (RNA and proteins) from the various cell types it is made of. Therefore, there is a high risk that changes in the expression of genes involved in MEC functions could be masked by their expression in surrounding cells. For example, genes involved in lipid biosynthesis are expressed in MEC and adipocytes but not regulated in the same way during lactation [14,15].

Therefore, to accurately and reliably follow molecular changes occurring in MEC for comparison purposes between physiologically different stages and genetically or environmentally perturbed systems, it is necessary to isolate MEC preserving biomolecule (RNA and proteins) integrity.

Different techniques, such as immunomagnetic separation [16-19], cell sorting [20] and tissue-depletion [15] have been used to isolate more or less homogeneous populations of MEC from milk or mammary tissue. MECs isolated from milk are easy to collect non-invasively and constitute a valuable source of material for analyzing mammary transcript profile during lactation. Although it has been claimed that milk MECs reliably reflect the activity of the mammary epithelium in goats and cattle [21,22], one can expect that cells out of their physiological context and faced with stressful purification protocols very likely induce adaptive changes modifying their expression profile. Differentially expressed membrane antigens have been used to flow-sort viable luminal epithelial and MMC from freshly disaggregated adult virgin rat mammary parenchyma [23].

Another means to obtain MEC homogeneous populations is from cell culture. However, one major obstacle to molecular biological studies of MEC is the lack of established cell lines that secrete, or can be induced to secrete, fat globules and milk proteins [24]. While culture systems have helped to identify some of the factors controlling growth [25,26], morphogenesis [27,28], functional differentiation [29] and tumorigenesis [30,31] of the rodent mammary gland, the heterogeneous cellular composition of primary cultures derived from the intact mammary parenchyma [32,33] complicates the interpretation of responses *in vitro*. In addition, it is well-established that MEC in culture are subjected to dedifferentiation [34].

Laser Capture Microdissection (LCM), first described by Emmert-Buck *et al*. [35] is now well established as a powerful tool for isolating cells of interest under morphological control from heterogeneous tissues. Major issues that should be addressed when using such a sorting approach are the amount and integrity of biological material extracted for reliable subsequent analyses of biomolecules (DNA, RNA and proteins). Amplification of nucleic acids is still possible as well, provided integrity is preserved. RNA degradation remains one of the main concerns since it can extend dramatically, depending upon the tissue. Also, it may significantly impact gene expression profiling. Frozen tissues are recommended for RNA recovery.

Nevertheless, LCM is an appealing technique, but it introduces additional methodological hurdles, including tissue handling (fixation, storage and staining) and maintenance of molecular integrity. The success of a microdissection experiment first depends upon the ability to distinguish cell types of interest from their morphological features. Immunological labelling may be required and used to assist in the identification of cells. In other words, if gene expression experiments are targeted, the challenge is to design a global protocol ensuring acceptable tissue morphology to facilitate isolation of cells while preserving accessibility and integrity of RNA, keeping in mind that this is critically tissue-dependent.

Successful application of LCM in transcriptomic analyses relies upon three critical factors: good tissue morphology, capture efficiency, and maintenance of RNA molecular integrity. Effective balancing of these three factors is required to recognize regions and obtain reliable transcriptomic results. Since ruminant mammary gland is one of the richest tissues in RNAse activity, classical protocols require accommodation to preserve RNA from RNase (endogenous and exogenous) and keep it intact in captured cells. This study was carried out to address these issues with the aim of developing a convenient and reproducible protocol to isolate MECs from ruminants (goat, sheep and cow) lactating mammary gland, preserving tissue morphology and RNA integrity, to develop a comprehensive overview of the genome expressed in MECs in their physiological environment.

Given that recent studies [36-38] reported the effects of tissue manipulation on RNA quality and gene expression, and that each tissue requires specific protocol for reliable results, we have evaluated the impact of the main critical steps (sampling, freezing, cryosectioning, staining, dehydration, and microdissection) during slide preparation and capture of MEC. In addition, we examined selectivity of this technique in evaluating enrichment in MEC as well as contamination by other surrounding cell types such as MMC, and immune cells (macrophages and lymphocytes) using qPCR.

## Methods

### Animals and tissue collection (sampling)

Surgical and experimental procedures were performed in compliance with the policies of INRA's Animal Care Committee. Mammary tissue was sampled from 5 goats, 2 ewes and 1 cow euthanized under safe and painless conditions, at the middle of lactation after milking and slaughtering. To preserve morphology and RNA quality we applied two different methods of freezing (liquid nitrogen or cold isopentane) immediately after collection with and without embedding medium as follows: the collected tissue was washed in cold PBS solution, 3-5 mm pieces of tissue were cut 3 and embedded in OCT^® ^(TissuTek™) in a cryomold of 1 cm^3 ^(Bayer™) and immediately immersed in liquid nitrogen or in SnapFrost™ system (Alphelys, France) containing cold isopentane at -80°C. Alternatively, some pieces were directly introduced in empty 1.5 ml cryotubes and immediately frozen in the same way (liquid nitrogen or SnapFrost™ system). Samples were stored at -80°C until further processing. The time delay between slaughtering and tissue freezing was less than 20 minutes.

### Slide preparation: cryo-cutting and dehydration

Frozen tissue blocks were mounted on the cryostat stage (Thermo Shandon, France) set at -20°C. Before transfer, the working environment was treated to be RNAse free and glass slides (uncoated, LLR2-45, CML, France) were pre-cleaned with RNAse Zap™ (Ambion, Applied Biosystems^®^) and rinsed in three baths of distilled water before a final bath in 70% ethanol. To test whether the effect of slide temperature plays a key role in detachment of MEC from glass slides during the laser-capture process, pre-cleaned slides were chilled (on ice or at 4°C) or not (room temperature) before transfer. Manufacturers' and published protocols recommend cutting 5 to 12 μm section thicknesses. Tissue sections were 8 μm thick, a compromise to ensure an optimal RNA yield preserving morphology as well as dehydration and laser-capture process efficiency.

Only one section was mounted on each apposing slide. To assess the possibility of conserving slides (few hours to several days) after section transfer, two different methods of cold storage were tested: after cutting, the slides were immersed in cold 75% ethanol and placed at -20°C or put directly into a tube containing desiccant and stored at -80°C. To avoid RNA degradation, each step of the slide preparation process was performed as quickly as possible. Water was RNAse free. Bottles of absolute ethanol (SIGMA-ALDRICH, France) and M-Xylene, anhydrous, >99% (SIGMA-ALDRICH, France) were opened just before use to dehydrate a maximum number of 8 slices a day to ensure proper dehydration. Sections were stained using Histogene^® ^staining solution (Arcturus, Applied Biosystems^®^) or Cresyl violet^® ^(LCM staining kit Ambion, Applied Biosystems^®^). Different protocols for dehydration and staining of frozen mammary sections were compared (Table 1). They were assessed looking at morphology and RNA integrity. After dehydration, slides were kept dehydrated 15 min or more (max 3 h) in a vacuum.

**Table 1:** Different protocols tested to optimize tissue section preparation before laser capture microdissection of mammary epithelial, yield and integrity of RNA extracted from microdissected cells.

	*N°1*	*N°2*	*N°3*	*N°4*	*N°5*	*N°6*	*N°7*	*N°8*
Ethanol 95%						30 s		

Ethanol 75%	30 s	30 s	30 s	30 s	30 s	30 s	**60 s**	30 s

Ethanol 50%						20 s	**20 s**	

RNA protector			RNA later^®^ 100 μl, 30 s	RCL2^®^ 100 μl, 30 s	RCL2^®^ 100 μl 30 s			

Water	+		30 s	30 s				+

Staining	HistoGene^®^ 15 s	HistoGene^®^ 10 s	HistoGene^®^ 10 s	HistoGene^®^ 10 s	Cresyl Violet^®^ 20 s	Cresyl Violet^®^ 20 s	**Cresyl Violet^®^** **20 s**	-

Ethanol 50%						20 s	**5 s**	

Water	+							+

Ethanol 75%	30 s	5 s	5 s	5 s	5 s	30 s	**30 s**	30 s

Ethanol 95%	2 × 1 m	2 × 1 m	2 × 1 m	2 × 1 m	2 × 1 m	30 - 40 s	**2 × 1 m**	2 × 1 m

Ethanol 100%	2 × 1 m	2 × 1 m	2 × 1 m	2 × 1 m	2 × 1 m	30 - 40 s	**2 × 1 m**	2 × 1 m

Xylene	2 × 5-10 m	2 × 5-10 m	2 × 5-10 m	2 × 5-10 m	2 × 5-10 m	2 × 5-10 m	**2 × 5-10 m**	2 × 5-10 m

Time of LCM	30-40 m	30-40 m	30-40 m	40-50 m	40-50 m	40-90 m	**40-90 m**	30 m

RNA integrity (ΔRIN)	-3.5 to -6	-3 to -4	- 2	-2 to -3	-0.5 to -1	-0.5 to 1	**- 0.5 to -1**	- 2

Morphology	++++	+++	----	+++	+++	++	**+++**	

In protocols 1 to 4 tissue sections were stained using the HistoGene^® ^LCM frozen section Staining Kit (Arcturus). Cresyl Violet^® ^staining (protocols 5 to 7) using Ambion LCM staining Kit provides good morphology and yields high quality RNA. The best protocol was determined to be No.7 (framed and bold), because of the high RNA integrity and morphology and because it was easier to handle without any RNA protector and quicker to perform.

To preserve the quality of RNA during staining, without ruining the morphology, we tried to add RNA protectors (enzymatic or chemical) used commonly to block RNAse activity, before HistoGene^® ^staining. Two chemical protectors were used: RNA later^® ^(Ambion, protocol N° 3) or RCL2^® ^(Alphelys, France, protocols N° 4 and 5), a new fixative which preserves morphology and nucleic acid integrity. In protocols N°3 to 5, 100 μl of RCL2^® ^or RNA later^® ^were dropped on the tissue section just before staining, to react for 30 s and removed by tapping the slide on an absorbent paper. RNase out (Invitrogen, Applied Biosystems^®^) or RNAsin (Promega) were prepared (2.5 μl at 40 U/μl, in 100 μl final of HistoGene^®^) and added on slice for 15 s during staining step.

### Laser Capture Microdissection

The LCM process was carried out using the Veritas Microdissection Arcturus system and software (Applied Biosystems^®^). Capture, which is the gentlest technique and thus maximizing biomolecules integrity, was performed under 40× or 100× magnifications using CapSure LCM macrocaps (Arcturus, Applied Biosystems^®^). IR Laser setting was chosen to maximize the size of the laser spot in the middle of alveoli (luminal side in which MEC are arranged in a monolayer epithelium) without contaminating the sample with non-target-tissue (MMC or interlobular stroma). Laser setting ranged between 75 to 90 mW in power, 1,300 to 3,500 μsec in duration, and 200 mV in intensity. Efficiency of microdissection was evaluated by examining the cap after capture and the tissue section remaining on the slide before and after lifting off the cap: if necessary, the non target tissue can be removed directly on the cap by lower power UV laser (2-5 mW).

The critical time limit for capture was estimated by examining RNA integrity and was evaluated from 30 minutes to 2 hours. The corresponding target area was between 300,000 and 600,000 μm^2 ^(around 150 to 200 acini).

### RNA extraction

Total RNA was extracted from captured cells using the PicoPure^® ^RNA Isolation Kit (Arcturus, Applied Biosystems^®^) according to the manufacturer's instruction protocol, including on-column RNase-free DNase I treatment (Qiagen S.A.- France, Courtaboeuf). CapSure macrocaps with captured cells were inserted into RNase-free 500 μl microcentrifuge tube containing 25 μl of extraction buffer (XB). The tubes were inverted to allow the reaction between the buffer and the surface of the cap. RNAs were extracted from scraped sections (tissue remaining on the slide after capture) by pipetting 50 μl of XB buffer onto the remaining tissue on the glass slide and gently scraped off and transferred in RNase-free 500 μl microcentrifuge tube. RNAs from cap and section scrapes were eluted respectively with 15 μl and 30 μl of elution buffer (EB). To assess RNA quality of tissue before manipulation, one cryo-section of mammary tissue was immediately treated to extract RNA using the same protocol as that for section scrapes after LCM.

### RNA quality control and cDNA synthesis

Purity, concentration and integrity of total RNA isolated in this manner were assessed using two independent techniques. RNA purity was evaluated by absorbance readings (Ratio A260/A230 and A260/A280) using the NanoDrop ND-1000 spectrophotometer (Thermo Fisher Scientific, Wilmington, DE). The fluorimetric method and micro-capillary electrophoresis device developed by Agilent Technologies was chosen to determine RNA concentration and quality with RNA 6000 pico LabChip Kit in the Agilent Bioanalyzer 2100 system. Quality was evaluated using the RNA Integrity Number (RIN) value introduced by Agilent [39].

First-strand cDNA was synthesized from 5-10 ng total RNA primed with oligo(dT)_20 _and random primers (3:1, v/v) using Superscript III reverse transcriptase (Invitrogen, Applied Biosystems^®^) according to manufacturer's instructions. Then, 1 μl of RNase H (2 U/μl, Invitrogen) was added and incubated 20 min at 37°C to remove RNA. The obtained cDNA was stored at -20°C before qPCR.

### Determination of MEC enrichment by LCM using qPCR

To estimate MEC enrichment obtained after LCM, *CSN1S2 *and *LALBA *transcripts, two specific markers of MEC, were quantified using qPCR (SYBR Green chemistry). Two internal control genes, S24 ribosomal protein (*RPS24*) and cyclophylin (*PPIA*) were quantified for accurate normalization of data [40].

Primers used were previously described [7,40] and qPCR systems were designed to quantify specific markers for MMC (*Krt14*), lymphocytes (*CD3e*) and macrophages (*CD68*). We also quantified transcripts from Fatty Acid Synthase (*FASN*) which is expressed in several cell types including MEC and adipocytes.

Comparing the structural organization of encoding genes across species (human, mouse and ruminants) and mRNA sequences at exon-exon junctions, we identified highly conserved regions on which primer pairs were designed, using Primer Express Software, version 2.0 (Applied Biosystems^®^). Primers were designed and purchased from Eurofins Genomics (France) to amplify goat, sheep and cow genes (Table 2). Amplification reactions were run (in triplicate) on an ABI PRISM 7900HT Sequence Detection System (Applied Biosystems^®^). First, primers efficiency was validated with a standard curve of four serial dilution points of a scraped section cDNA pool (ranging from 1000 pg to 1 pg of total RNA reverse transcripts), and a no template control (NTC). qPCR amplification mixture (20 μL) contained 5 μL single strand cDNA template diluted 4 times after reverse-transcription, 10 μL 2× Power SYBR Green PCR Master Mix buffer (Applied Biosystems^®^) and 1.2 μL forward and reverse primers (5 μM) to reach a final primer concentration of 300 nM. After optimization of qPCR systems (efficiency -3.32 to -3.4), we used the ΔΔCt method and RQ Manager Software (Applied Biosystems, version 2.3), as well as in the relative expression software tool (REST 2009 V2.0.13^©^, Qiagen), to compare expression of each gene between captured cells and its scraped tissue, following the MIQE guidelines [41].

**Table 2:** Primers used in this study.

RPS24 F	TTT GCC AGC ACC AAC GTT G
RPS24 R	AAG GAA CGC AAG AAC AGA ATG AA

PPIA F	TGA CTT CAC ACG CCA TAA TGG T

PPIA R	CAT CAT CAA ATT TCT CGC CAT AGA

CSN1S2 F	CTG GTT ATG GTT GGA CTG GAAAA

CSN1S2 R	AAC ATG CTG GTT GTA TGA AGT AAA GTG

Krt14 F	CCC AGC TCA GCA TGA AAG C

Krt14 R	AGC GGC CTT TGG TCT CTT C

CD3e F	ACG CTGT ACC TGA AAG CAA GA

CD3e R	AAT ACA CCA GCA GCA GCA AG

CD68 F	GAT CTG CTC TCC CTG AAG CTA CA

CD68 R	CAT TGG GAC AAG AGA AAC TTG GT

FASN F	ACA GCC TCT TCC TGT TTG ACG

FASN R	CTC TGC ACG ATC AGC TCG AC

Each pair of primers amplifies the target cDNA in its 3' region. Primer pairs were designed with the Primer Express Software v2.0 (Applied Biosystems) except for RPS24 primers which were manually designed.

### Statistical analysis

Reliability of reference genes (*RPS24 *and *PPIA*) was evaluated with GeNorm Visual Basic application for Microsoft Excel as described by [42].

The relative expression for each gene of interest between caps versus scraped tissues was tested for significance by a randomized test implemented in the relative expression software tool (REST 2009 V2.0.13^©^, Qiagen), based on Pair Wise Fixed Reallocation Randomized Test^© ^[43].

## Results and discussion

The main concerns when using LCM to analyse gene expression of a specific cell type is first to efficiently and selectively capture the right cells and second to obtain RNA of good quality. To address these issues and to optimize a LCM experimental design for mammary tissue which is highly heterogeneous and rich in endogenous RNase, a systematic approach was undertaken to evaluate the impact of different critical steps and parameters from tissue sampling and freezing to dehydration (essential when using capture technology) on cell isolation and RNA yield and integrity.

### Freezing conditions and tissue morphology

In this study, *ca*. 150 slides of tissue sections were cut from 8 mammary glands taken on three different ruminant species: goat, sheep and cow. The impact of the different steps of slide preparation was evaluated and we observed that the early steps, mainly sampling, freezing, cutting and staining of tissue, play a crucial role for a consistent success in capture of alveoli MEC from mammary sections.

**Figure 1:**
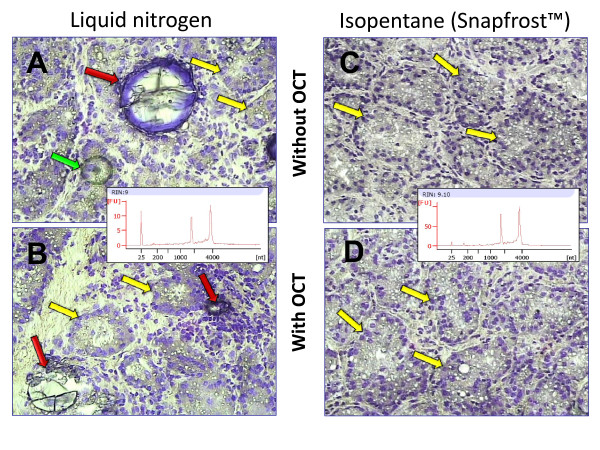
**Impact of the freezing system on morphology of fresh mammary tissue sections and on quality of RNA extracted**. Alveolar (acini) structures lined by MECs (yellow arrows) can be easily distinguished after staining mammary tissue sections with Cresyl violet AMBION. Immediately after collection, mammary tissue samples were washed in cold PBS solution, cut in cube of 3-4 mm thickness, and frozen in four different conditions: pieces of mammary tissue were either directly introduced into 1.5-ml eppendorf tubes and frozen in liquid nitrogen (A), or in cold isopentane (-80°C) using the SnapFrost™ system (C), or embedded in OCT^® ^contained in cryomold before to be immediately immerged in liquid nitrogen (B) or in cold isopentane (D). RNA quality (RIN) which was estimated by RNA 6000 Pico LabChip kit and Agilent 2100 Bioanalyzer, was identical (ranging between 8.5 and 9.5) whatever the freezing procedure, as illustrated in the electrophoreris profiles. Some large blisters (red arrows on Figure A and B) appear however in biopsy flash frozen in liquid nitrogen, mainly without cryoprotector, and morphological details are better seen on tissues frozen using the SnapFrost™ system (Magnification: ×60). The green arrow indicates the thermoplastic film stuck on epithelial cells to be captured.

Morphology of mammary tissue and RNA quality (RIN value) obtained with or without OCT^® ^using two different frozen conditions, liquid nitrogen and cold isopentane, are shown in Figure 1. Whereas RNA integrity was preserved under both conditions (RIN for scraped tissue ranged between 8.5 and 9.5), morphology of mammary sections frozen in isopentane using the SnapFrost™ system (Figure 1C and 1D) was better than for tissue frozen in liquid nitrogen (Figure 1A and 1B). Total immersion of OCT^®^/tissue/cryomold in liquid nitrogen results in loss of morphological details (Figure 1B) and some morphological artifacts appear in mammary tissue compared to immersion in isopentane (Figure 1C). Rapid freezing in isopentane at -80°C is commonly recognized to provide good morphology and molecular preservation mainly because -80°C is a temperature low enough to prevent the formation of large crystals damaging tissues. In addition, contrary to liquid nitrogen, isopentane does not outgas violently in contact with tissue samples and thereby eliminates the risk of fractures which are opportunities for immediate and long term degradation during storage and at thawing. Total immersion of OCT^® ^embedded tissues into liquid nitrogen resulted in cracked OCT^® ^and formation of bubbles within the specimen.

This phenomenon is amplified when tissue is frozen without OCT^® ^in liquid nitrogen (Figure 1A). Large morphological artifacts such as scratch marks or blisters are observed and impaired correct IR laser impacts during LCM. Consequently, a precise capture due to a different distance between the bottom of cap and tissue section became difficult. In contrast, the SnapFrost™ system which is a cryo-bath allowing a control temperature of isopentane (-80°C), reduced freezing-fixation artifact: blocks showed a good morphology and mainly a reliable quality of tissue.

### Thickness of Cryo-sections, slide temperature

**Figure 2:**
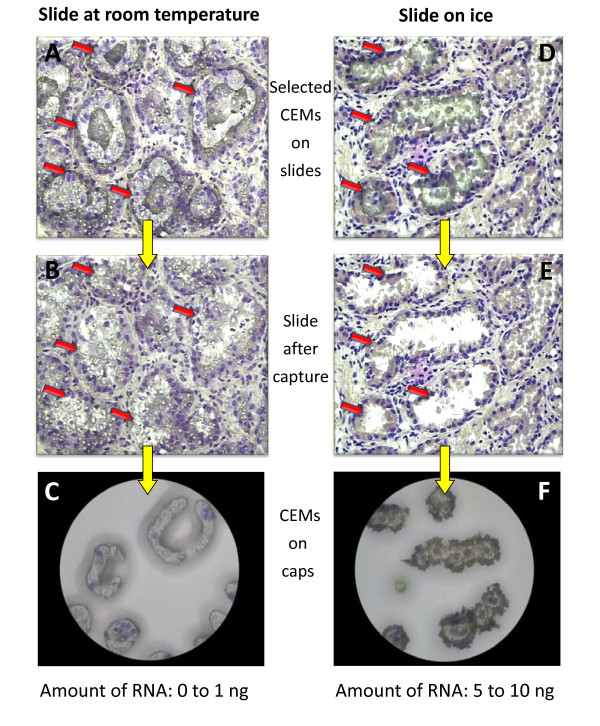
**Impact of glass slide temperature on cell transfer efficiency and RNA extraction yield**. Frozen tissues cut at 8 μm and transferred on glass slides that have been placed at room temperature (A) or chilled and kept at 4°C, before transfer (D). As evidenced by the number of cells captured (C and F) and the RNA yield, given under each cap (C and F) the efficiency of capture was very low for the slides placed at room temperature. A/D: Cresyl violet^® ^stained slides before LCM; red arrows indicate the thermoplastic film stuck on epithelial cells to be captured. B/E: mammary tissue section remaining on the glass slide after LCM. C/F: microdissected mammary epithelial cells transferred on the LCM cap. Magnification: ×60.

Tissue was cut at 6, 8 and 10-μm thickness. The best compromise for obtaining a sufficient amount of material while preserving tissue morphology was 8-μm thickness. Temperature of the slide before sectioning and transferring the tissue section on the slide also appeared to be critical (Figure 2). Whatever the protocol subsequently applied for slide fixation and staining an optimal temperature difference of 20-25°C between the cryostat and the glass slide is required to succeed in capture. The most efficient transfer was obtained by keeping slides on ice or in a refrigerator before use. This parameter seems to be crucial since the amount of RNA extracted can reach up to a 15-fold increase, varying between 10-15 ng for cold slides vs. 0-1 ng for slides kept at room temperature.

It is difficult to evaluate the exact number of MEC isolated after 40 to 60 min of LCM since this it depends on the number of acini present on the slide and on the MEC per acini (10 to >40). Given that the Veritas Arcturus system allows quantification of captured material via a tool estimating the total area selected before capture, we can establish a correlation between captured area (mm^2^) and RNA quantity. Usually, with goat and cow samples, we get 5 to 10 ng of RNA per cap (*ca*. 300,000 to 600,000 μm^2 ^of captured cells, corresponding to more or less 2500 cells: 150 alveoli × 25 cell sections in average per acinus). In other words, there are 3 picog RNA in 1/3 cell since we work on 8-μm tissue section thickness, and therefore one can estimate to *ca*. 10 picog the amount of RNA contained in one MEC. However with sheep, the amount of material we obtained was between 1.5 and 2 times higher.

### Storage conditions of slides

We also tested the possibility of keeping the slide with tissue sections in cold 75% ethanol at -20°C in order to find conditions stabilizing mammary tissue sections for a long period, before LCM treatment. We observed that in this way we did not impact capture efficiency and stabilized tissue sections from few hours before treatment to several days, even one week. We also tested whether tissue sections on slides can be stored at -80° C for several days. Slides were put into 50 ml Falcon tubes with desiccant, quickly placed in dry ice and stock at -80°C. We observed that morphology and RNA quality were not affected although the quantity of captured material was always very poor. In conclusion, we choose to put slides in cold 75% ethanol at -20°C until the staining step.

### Staining and dehydration impact on RNA yield and integrity

The next step was to evaluate the impact of fixation, staining and dehydration on RNA integrity and yield as well as on tissue morphology. Previous studies [37,38,44] have shown that these steps are crucial for obtaining good and reproducible results, regardless the kind of tissue. We compared two staining conditions commonly used for LCM studies (Ambion, LCM staining kit with Cresyl Violet^® ^stain solution and Arcturus HistoGene^® ^kit with Histogene stain solution) and different times of dehydration. HistoGene^® ^stain is a special solution developed by Arcturus to stain tissues for LCM subsequently used as sources of RNA. It is a fast penetrating stain that provides good contrast by differential staining of nuclei (purple) and cytoplasm (light pink). Cresyl Violet^® ^is a hydrophilic, basic stain that binds to negatively charged nucleic acids without water step during slide preparation to re-hydrate the tissue.

**Figure 3:**
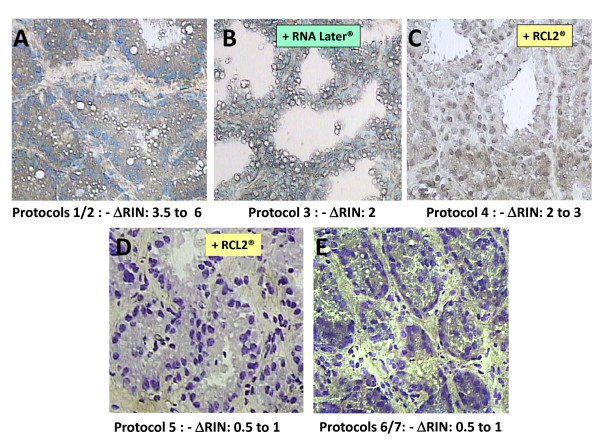
**Impact of histological stain and fixative/protector on morphology of goat mammary tissue and RNA integrity**. Frozen mammary tissue sections were stained either with HistoGene^® ^(A to C) or with Cresyl violet^® ^(D and E). MEC isolated after treatment with RCL2, a new fixative preserving tissue morphology and Nucleic Acid integrity (C and D), or with RNA later (B) provide RNA of better quality, expressed as ΔRIN which is difference between the RIN value obtained with total RNA extracted from the mammary tissue section (8.5) and the RIN value obtained with RNA extracted from microdissected cells.

Results are given in Table 1 and Figure 3. The best morphology was obtained with HistoGene^® ^following the protocol recommended by Arcturus, (N°1) which easily distinguished the cytoplasm (stained in brown) and the nucleus (stained in blue, Figure 3A). However, we observed significant variations in RNA quality (RIN value ranging between 6.5 and 3) across serial slides prepared from the same bloc of tissue whereas the RIN value obtained with RNA extracted from the whole tissue was 8.5. To reduce RNA degradation, we eliminated water steps before and after staining (protocol N°2). Stain penetration was then lower, allowing however MEC to still be easily recognized, and limiting RNA degradation significantly as compared with protocol N°1 although RNA quality was still non reproducible. We hypothesized that HistoGene^® ^can accelerate degradation by reactivation of endogenous nucleases present in mammary gland, likely due to pH of HistoGene^® ^solution (pH measured ~4.0), whereas neutral pH of Cresyl violet^® ^solution (pH measured ~7) avoids degradation of RNA. After substitution of HistoGene^® ^by RNase-free water (protocol N° 8), RNA degradation was reduced by less than one RIN unit. However, it was obviously not possible to recognize cells on unstained tissue sections. Given these results we decided to use the Cresyl violet^® ^as stain for further experiments.

Dehydration is crucial to stabilize the tissue and to allow capture. All the steps following dehydration (identification and selection of areas of interest, adjusting laser parameters and capture) are time consuming and it is necessary to keep tissues dehydrated to avoid degradation of RNA. A high ambient humidity could rehydrate the tissue and reactivate endogenous RNases. In addition, moisture impedes film adhesion and cell capture. For these reasons, we limit LCM to 45-60 minutes which proved to be a good compromise to overcome the variations in relative humidity. Ordway *et al*. [45] recently showed the detrimental effect of relative humidity of the laboratory where tissue sections are stained, handled, and submitted to LCM, thus impacting the performance of the instrument and the quality of RNA extracted from tissue sections. Low relative humidity in the laboratory (lower than 23%), was conducive to little or no degradation of RNA extracted from tissue following staining and fixation and to high capture efficiency by the LCM instrument. Clément-Ziza *et al*. have proposed to perform LCM under an argon atmosphere, thus preventing tissue rehydration to finally stabilize RNA [44]. These authors have also assessed several staining solutions in regard of their effect on tissue morphology and RNA integrity. They have noticed that stains that were very efficient when dissolved in water, such as hematoxylin and eosin B, are faint or poorly resolutive in alcoholic solvent. They also observed significant RNA degradation when alcoholic staining solutions containing hematoxylin were used. On the other hand, the cresyl violet in ethanolic solution seems to be appropriate to perform LCM experiments as shown in our study on the mammary tissue.

### Fixatives and RNA protectors: impact on Yield and Integrity of RNA

Following these results, we hypothesized that addition of RNA protectors (RNase inhibitors) such as RNA later^® ^(protocol N° 3) or RCL2^®^, are promising new noncrosslinking fixatives [46], preserving morphology and nucleic acid integrity (protocols N° 4) before HistoGene^® ^staining and could improve RNA quality. Actually, RNA degradation was especially reduced with RNA later^® ^(loss of one RIN unit) whereas with RCL2^® ^we observed a loss of 1.5 to 2.5 RIN units. However, RNA later^® ^provokes a loss in morphology (Figure 3B, protocol N° 3) compared with RCL2^® ^(Figure 3C, protocol N° 4). Therefore, RCL2^® ^provides a good compromise to get morphology and RNA quality. The same test was carried out with Cresyl violet^® ^(Figure 3D, protocol N° 5). We found that this stain did not affect RNA integrity, and addition of RNA protector was without any effect. In fact, with or without protector, loss of RIN units was less than 1. Morphologically, the acini were well identified with Cresyl violet^® ^and RCL2^® ^slightly improved the image.

RNasin (Promega, France), a potent inhibitor of ribonucleases, was also tested prior to staining since such inhibitory activity was reported to protect RNA (included in staining solution) from degradation in LCM experiments for gene expression profiling of basal cell cancer tissues [47,48]. We did not observed any significant improvement in quality (RIN scores) of RNA recovered from mammary tissue sections treated in such a way.

Nevertheless, there was no considerable improvement in either quality or quantity of RNA recovered from the tissue sections after an inhibitor treatment. In conclusion, we finally decided to opt for protocol N° 7 (without any RNA protector) which is easier to handle and quicker to perform.

### MEC enrichment by LCM: contamination by MMC and immune cells

qPCR experiments were performed on reverse transcribed RNA extracted from microdissected cells targeting specific gene transcripts to evaluate enrichment in MEC.

To determine potential cell contamination of the laser-captured cells by adjacent MMC or to estimate the selective capture of MEC, mRNA transcript levels of cell-specific markers were assessed by evaluating the relative expression between captured cells and their corresponding mammary tissue scrapes, after LCM. Relative quantity with specific markers for MMC (*Krt14*) and MEC (*LALBA *and *CSN1S2*) was assessed after normalization, using *PPAI *and/or *RPS24 *as reference genes. *FASN*, a gene expressed in a large panel of tissues [49] including several cell types mainly in cells with high lipid metabolism such as adipocytes, hepatocytes but also in fetal proliferative epithelial cells and MEC, was also assessed under the same conditions. Levels of *LALBA *(RQ mean = 1.47), *CSN1S2 *(RQ mean = 1.33) were significantly increased. *FASN *was relatively unchanged (RQ mean = 0.85), suggesting that active fatty acid synthesis which is required for energy utilization and membrane synthesis is equally expressed in MEC and in the surrounding tissue. These results attest to the enrichment in MEC after LCM even though increasing in *LALBA *and *CSN1S2 *transcripts are less striking, given the high percentage of MEC (around 80-90%) and the low number of other cell types in lactating mammary parenchyma sections.

**Figure 4:**
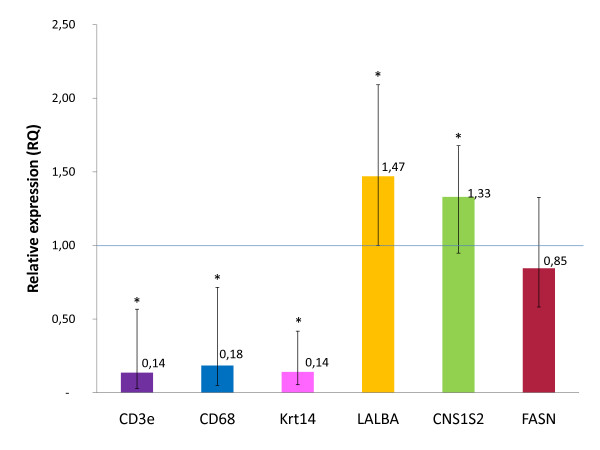
**Selectivity of MEC capture assessed by real-time quantitative PCR**. To estimate contamination of the laser-captured MEC by adjacent MMC as well as by immune cells (macrophages and lymphocytes) mRNA transcript levels of cell-specific markers were assessed by measuring the relative expression of the relevant genes between captured cells and mammary tissue scrapes, i.e. the mammary tissue remaining on slides, after LCM. Relative quantities of specific markers from MMC (*Krt14*), macrophages (*CD68*), lymphocytes (*CD3e*) and MEC (*CSN1S2*, *LALBA*) were assessed after normalization (*PPAI *and/or *RPS24*). *FASN*, a gene expressed in a large panel of cell types, including adipocytes and MEC, was also assessed in the same conditions. Mean RQ values are given for each gene. *Krt14 *decreased (RQ = 0.14; *ca*. 7-folds reduction) in the captured cells compared with the whole mammary tissue. Likewise the same ratio was observed with *CD68 *(RQ mean = 0.18) and *CD3e *(RQ mean = 0.14). Levels of *LALBA *(RQ mean = 1.47 fold), *CSN1S2 *(RQ mean = 1.33 fold) were significantly increased. *indicates ratio significantly different between cap and scraped tissue (*p *< 0.001).

Significant results were recorded when quantifying messengers from genes specific for other cell types such as MMC and immune cells. Thus, levels of *Krt14 *messengers decreased dramatically (RQ = 0.14; *ca*. 7-folds reduction) in the captured cells compared with the whole mammary tissue. A weak expression of *Krt-14 *was systematically observed in microdissected MEC, reflecting a slight contamination by MMC during capture. This is due to a very close proximity between MMC and MEC [50,51] as shown in confocal images of a breast section double-stained for both cell types where double-stained suprabasal cells are occasionally found [52]. Similar results were obtained with *CD3e *(RQ mean = 0.14), a marker of lymphocytes and *CD68 *(RQ mean = 0.18) which suggests the putative presence of macrophages (Figure 4), further demonstrating the efficiency of LCM.

High standard deviations were observed for these qPCR experiments, pointing out the degree of confidence that can be given to the results in terms of statistical conclusions. It must be kept in mind that gene expression measurement techniques such as qPCR not only require a normalization strategy to allow meaningful comparisons between biological samples [53], but also demands work with RNA of good quality (RIN > 7), in sufficient amount and that genes for which the expression is measured must be expressed at a sufficient level.

Typically, all these parameters have to be considered and the first one is usually accomplished through the use of endogenous housekeeping genes that are presumed to show stable expression levels in the samples under study. Which specific genes and how they can be measured in limited amounts of mRNA such as those extracted from microdissected cells still remains a concern. GeNorm software confirmed that *PPAI *and *RPS24 *are actually highly reliable reference genes for normalization purposes. To calibrate input amounts of starting material, cell count and/or total RNA are useful but they are not precise enough and reliable enough to serve as normalization standards. Demonstrating relative enrichment in one cell type after microdissection is difficult since we start from heterogeneous tissues. Therefore, what calibrator (tissue scrapes or whole tissue) to use to perform relative gene expression measurements made by qPCR? We chose to use isolated cells (each cap) against all scrape sections since it can be considered as a mean value of serial tissue sections.

## Conclusions

LCM makes it possible to obtain highly MEC enriched material from lactating mammary tissue sections preserving RNA integrity. In addition, we provide molecular evidence for successful selectivity of the capture method despite the difficulty of disassociating luminal secretory cells (MEC) from MMC bordering the basal lamina which separates the epithelial layer from the extracellular matrix.

To accurately evaluate the expression of genes specifically expressed in MEC, such as those encoding LALBA and CSN1S2 represents a step forward in determining the transcriptional profile of a cell type and how it can be modulated by environmental factors such as feeding, stress, milking frequency, the health status or gene polymorphisms. In addition, to understand how genes expressed in several cell types are regulated, it is crucial to work on pure or at least enriched cell populations. Otherwise, expression analyses could potentially lead to artefactual results. This is well-exemplified by *FASN*, a gene encoding the fatty acid synthase which is widely expressed in many tissues and cell types, including the alveolar secretory epithelium and adipose tissue. Relative proportions of these tissues, both involved in lipid metabolism, dramatically change during pregnancy.

Capturing other cell types or stroma provides the opportunity to examine further to understand the mechanisms involved in growth regulation and morphogenesis of the mammary gland. Until now, most attention has been paid to the luminal epithelial cell which is the functionally active milk-producing cell and the most likely target cell for carcinogenesis. However attention on myoepithelial cells has begun to evolve with the recognition that these cells play an active part in branching morphogenesis and tumor suppression [54]. However, the major question remains to know how the luminal epithelial and myoepithelial lineages are related and how they arise from a common putative stem cell population. Xenotransplantation in mice and comparative studies across species (ruminants and humans), have shown that the fat pad plays a crucial role in mammogenesis [3]. Furthermore, it was observed in ruminants that during pregnancy, stroma would contribute to mechanisms that regulate growth. This could be extrapolated to other species, especially women and mice for which fat remains important, even during lactation. However, the greatest challenge remains to assess the contribution of local mechanisms that regulate growth which may explain the range in tumorogenic susceptibility of the mammary gland between species. Since information obtained from rodents may not always be directly transposed to the human breast and that ruminants show a morphology and mammary parenchyma development similar to humans, ruminants remain a pertinent model to go further into mammary gland biology understanding. Capturing different cell types makes it possible to establish cell specific gene expression profiles from healthy tissue, useful for discovering new tumor biomarkers.

## List of Abbreviations

**qPCR: **real time quantitative Polymerase Chain Reaction; **MEC: **Mammary Epithelials Cell; **MMC: **Mammary Myoepithelial Cell; **RIN: **RNA Integrity Number; **RQ: **Relative Quantification; **RPS24: **Ribosomal Protein S24; **PPIA: **cyclophylin A; **Krt14: **Cytokeratin 14; **FASN: **Fatty Acid Synthase; **CSN1S2: **α_s2_-casein; **LALBA: **α-lactalbumin;

## Authors' contributions

CB participated in study design, performed the LCM, RNA extraction and qPCR experiments, including statistical analyses, and drafted the manuscript. SM helped to perform the LCM and RNA extraction. JCH helped to perform qPCR and contributed to design qPCR systems. PD contributed to LCM protocol optimization. PM conceived the study and participated in its design and coordination and helped to draft the manuscript. All authors read and approved the final manuscript.
